# Assessment and Prediction of the Food Production Capacity and Consumption Gap in Arid Oasis Metropolitan Area

**DOI:** 10.3390/foods13244185

**Published:** 2024-12-23

**Authors:** Mingjie Cui, Yufang Zhang, Degang Yang, Wenqiang Xu, Rongqian Lu, Wenshuai Bai

**Affiliations:** 1 Key Laboratory of Ecological Safety and Sustainable Development in Arid Lands, Xinjiang Institute of Ecology and Geography, Chinese Academy of Sciences, Urumqi 830011, China; 2 University of the Chinese Academy of Sciences, Beijing 100049, China; 3 Key Laboratory of GIS and RS Application Xinjiang Uygur Autonomous Region, Urumqi 830011, China

**Keywords:** food security, food production, food consumption, Urumqi metropolitan area

## Abstract

Food security is the foundation of sustainable human development, and the balance between food supply and demand in urban areas is highly important for promoting residents’ health and the sustainable development of cities. This paper takes the Urumqi Metropolitan Area (UMA), a typical oasis urban area, as the study area and uses the food production—demand gap indicator to assess the balance of food production and consumption in the region from 2000 to 2020 and projects food demand in 2030–2060. The results show, first, that residents’ food consumption is characterized by high carbohydrate, protein, and fat consumption, and that this put more pressure on food production. Second, different food consumption structures will have different impacts on food production, and the local food production capacity in UMA falls short of ensuring a balanced nutritional structure for residents. Third, food demand increases significantly in 2030–2060, and the pressure of population consumption structure on food production is much greater than that of population growth. Considering the environmental effects of food transportation and the loss of food nutrients, on the production side, the construction of the UMA should be accelerated by including Qitai County and Jimsar County in the UMA’s planning scope, strengthening city—regional connections, and improving the local food production and supply capacity of surrounding areas. On the consumption side, regional dietary guidelines should be developed based on local dietary culture and agricultural production conditions to help guide residents to adjust their dietary structures, thereby alleviating pressure on local food demand. Such measures are crucial for ensuring sufficient food supply and promoting balanced nutrition among the population.

## 1. Introduction

With the acceleration of global urbanization, cities have become the main living centers of people. As populations continue to gather in cities, two-thirds of the population are expected to live in cities in the future [1]. While the World Cities 2022 Report noted a gradual slowdown in population growth, the population continues to increase, with urban populations expected to grow by 76 percent in low-income countries, 6 percent in upper-middle-income countries, and approximately 20 percent in high- and lower-middle-income countries by 2070 [2]. The production and consumption of food are also undergoing constant changes. Relevant studies have shown that, due to the increase in population income and population growth, global per capita meat consumption and total meat consumption are on the rise, especially poultry and pork consumption [3,4]. The population growth in China has a more far-reaching impact on food consumption, and the population growth in urban areas will have a more continuous impact on food consumption [5]. It is estimated that 80% of the world’s food will be sold to cities by 2050. With the continuous improvement of living standards and population growth, diet structure gradually becomes high quality and diversified, and the demand for food will increase significantly [6,7]. Although food production has generally kept pace with population growth, 800 million people worldwide still lack food, and the global food crisis has worsened since 2017 [8]. As the global population continues to migrate to cities and cities gradually expand, high-productivity farmlands are gradually being occupied [9], threatening the urban food production capacity.

Additionally, since the urban food system is jointly influenced by people, the environment, society, and the economy [6], almost all materials and energy in a city are dependent on external transportation [10]. Therefore, external environmental changes, such as extreme weather events, sudden risk shocks, and resource destruction [11,12,13], place greater requirements for the production of urban food systems, and the contradiction between urban food production and demand has become increasingly prominent. In this context, an increasing number of scholars have begun to study the food supply and demand balance. Some scholars have evaluated the production and demand of cereals, the demand of which is evaluated mainly through comprehensive assessments of rations, feed grains, industrial grains, seeds, and food waste and loss [14,15].

With people’s awareness of nutrition, scholars are paying attention to the supply and demand of food calories and nutrients. Song et al. used the land resource carrying capacity model to measure the balance between population, grain yield, and heat supply and demand [16]. Jensen and Orfila assessed the food production situation of Leeds using land-use methods. According to the total energy and nutrient requirements of residents of different ages and genders, there was a 51.6% gap between food production and demand in Leeds, England [17]. The above studies considered only the gap between the production and demand of staple foods, not the impact of dietary quality on the gap between production and demand.

Food diversity has long been regarded as a key element of a high-quality diet [18]. In China, the 2023 No. 1 Document of the Central Committee emphasized “establishing a big food concept and accelerating the construction of a diversified food supply system” [19]. Emphasis should be placed on the diversification and health properties of residents’ food according to the safety of staple food consumption and quantity [20]. Chen discussed the matching degree between the supply and consumption of agricultural products in China and reported a surplus in rations; however, there was a certain supply gap in feed, oil, sugar, meat, milk, and other foodstuffs [21]. Zhang et al. evaluated the balance between the supply and demand of grains, vegetables, meat, and fruits through the carrying capacity of land resources and reported that land resources in Tibet were characterized by an overall balance, local overload, and increased tension [22]. Although these studies considered the dietary diversity of residents, they did not consider the differences in residents’ food needs by age or sex.

As people increasingly realize the importance of food choice to the environment, an increasing number of studies have been conducted on “local food”, and the food self-sufficiency indicator is widely used. Ding et al. compared the production and demand of 12 kinds of food in various counties of the Qinghai—Tibet Plateau under different settings and reported that the region’s staple food supply could adequately meet future demand; however, it cannot meet the demand for feed grains [23]. After evaluating counties in the United States, Bingham et al. reported that most counties rely mainly on food circulation to meet local food needs [24]. Liang et al. assessed China’s food supply and demand over the past 30 years and reported that it would take more than the current arable land productivity to meet all food types [25]. Currently, most studies utilize the concept of food self-sufficiency to measure the current or future states of food self-sufficiency in China and other countries, and some research has also evaluated China’s food self-sufficiency at the provincial level. Although the concept of self-sufficiency is typically used to study large regions, it is equally applicable to smaller areas. Some scholars have conducted food self-sufficiency research at small regional scales [17]. Li et al. quantifies self-sufficiency in the eastern regions of China and showed that most areas could achieve self-sufficiency in grains, vegetables, and meat, but milk and soybeans were extremely scarce [26]. Wang et al. analyzed the food self-sufficiency capacity of the urban areas in China’s Pearl River Delta within an integrated agricultural land use framework and the results indicated that the food self-sufficiency capacity of this urban region is relatively low [27]. Many previous studies have assessed and predicted the supply and demand of current and future food systems, and they have focused mainly on large-scale regional studies in countries or regions or metropolitan areas. However, research on food self-sufficiency in oasis cities is lacking.

An oasis is a unique natural landscape, an important part of the ecosystem [28], and the most sensitive area for people and resources. A city is a geographical entity formed by the agglomeration of human social activities and is the most important place for human activity [29]. The special natural geographical background of an oasis in an arid region determines that its urban development depends on the spatial distribution of the oasis. An oasis city is the main form of urban development in arid regions, with the highest concentration of human activities. Group development is the basic pattern of oasis city development, mainly showing “great dispersion, small agglomeration” spatial distribution characteristics [30]. Better land and water resources in oases are the foundation for developing oasis agriculture. With the agricultural development of oases and the increase in the urban population, urban clusters with central cities at the core have gradually formed [31]. These oasis city clusters are hundreds of kilometers apart, showing closed and dispersed characteristics [32]. The Urumqi metropolitan area (UMA) is Central Asia’s largest urban cluster development area, with rapid urban development. It is also one of the main patterns of urbanization development in the arid region of Xinjiang, China. Its large population size and distinct ethnic characteristics are relatively rare among oasis cities and can best reflect the particularity of the interaction between human beings and oasis cities. UMA also has a special social status: it is an important node of the ancient Silk Road and is the bridgehead of China’s opening to the West. Its uniqueness is an important reason for this paper to use it as a case study. In addition, due to improvements in the level of urban development and increases in the population agglomeration effect, agricultural production has gradually decreased while food demand has gradually increased. Therefore, in the context of the high demand for food and limited production capacity in oasis cities, improving the food production capacity and resilience of the food system in oasis cities is key to the sustainable development of the urban food system in the UMA [33,34], which is also of great practical significance for maintaining the UMA’s social stability and ensuring residents’ life security. Measuring the food security of the UMA is central to this research. Therefore, we use a food self-sufficiency indicator to assess the gap between food production and demand. Several reasons support the use of this indicator. Firstly, achieving food self-sufficiency is a crucial prerequisite for ensuring food security, as it enhances the ability to withstand adverse events such as the outbreak of the COVID-19 pandemic in 2020. Secondly, a significant discrepancy between urban food production and demand can lead to an overreliance on food trade and long-distance transportation, resulting in substantial environmental costs [35,36] and degradation of nutritional quality [37,38]. Lastly, because agricultural production policies and crop layout planning are implemented at smaller regional levels, analyzing food self-sufficiency capacity at these levels provides a more comprehensive understanding of the region’s food system dependency and vulnerability. This understanding aids in devising precise urban agricultural production policies and in better aligning provincial and national food production and trade strategies, thereby contributing to national food security.

Existing studies on food production and demand lack consideration of food types, residents’ age and gender differences, and the particularity of oasis cities. Therefore, this study can fill in the gaps in the research on food production and demand and enrich the content in this field. In this context, we consider the changes in the consumption structure and pattern of urban residents in the UMA, analyze the gap between the production and demand of total calories for 9 kinds of food, and judge how the gap between total caloric production and demand occurs when food demand is guaranteed under different dietary structures. Second, the balance between the supply and demand of nutrients and food diversity under different dietary structures was further analyzed to identify the ability of food localization production to support regional residents’ food demand. Finally, the pressures and challenges of food demand from 2030 to 2060 are analyzed. Based on total calories, macronutrients and food diversity, our research provides a new research perspective for the construction of cities and the development of urban agriculture from the perspective of food supply and food demand security. Urban construction should not only focus on the quality of urban economic development, but also pay attention to the gap between urban agricultural production and population consumption demand, so that urban development can be more sustainable. Our purpose is as follows: 1. to understand whether the food production capacity in the UMA can guarantee food security and how the current maximum food production capacity could guarantee food security to increase people’s awareness of the local food production situation; 2. to clarify the ability of UMA to rely on local production capacity to ensure current and future food security in response to sudden crises or other situations; and 3. to put forward suggestions on oasis urban planning and oasis urban agriculture development from the perspective of food production, and put forward suggestions on promoting the transformation of residents’ food consumption patterns from the perspective of food consumption, so as to realize the transformation and upgrading of food consumption structure promoted by a food production structure and build a nutrition-oriented sustainable food system.

## 2. Materials and Methods

### 2.1. Study Area

**Figure 1:**
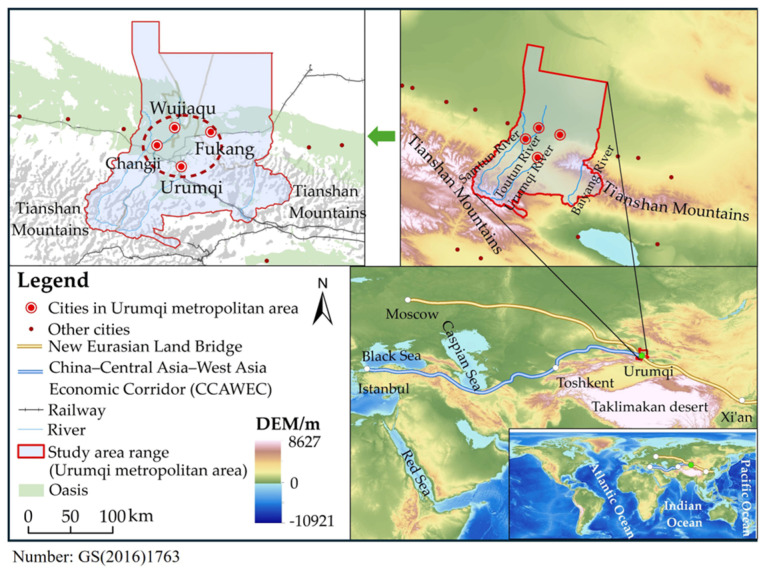
Study area (Global elevation models data source: GEBCO Compilation Group (2023) [40]).

The UMA is located in the arid area of northwest China, in the middle of the Tianshan Mountains and on the southern edge of the Junger Basin. It is located in the core area of the Tianshan Mountain North Slope urban agglomeration [39], the largest oasis urban community in Xinjiang and Central Asia, including Urumqi City, the 12th Division of the Corps, Changji City, and Wujiaqu City (Figure 1). It is the western bridgehead of the Chinese section of the New Eurasian Land Bridge and plays an increasingly important role in the economic development of western China and Central Asia. In the local deployment of the “14th Five-Year Plan”, the local government in the UMA vigorously promoted constructing a “one-hour economic circle”, gradually improved the urban integration between Urumqi and Changji, Wujiaqu, Fukang, and other cities in the circle, and enhanced the ability of the region to assume the strategic functions of the National Silk Road Economic Belt. In 2020, the total population reached 4,984,019, and the GDP reached 439.3 billion yuan in the UMA, accounting for 19% and 32% of the total Xinjiang population in the same period, respectively.

### 2.2. Data Collection

#### 2.2.1. Food Production and Demand Data

The food types involved in this study included mainly grains, tubers, legumes, vegetables, meat, aquatic products, eggs, milk, and fruit. The food production data are mainly from the statistical yearbooks of Xinjiang for the years 2000, 2010, and 2020 [41,42,43]. Data such as the demand and proportion of various types of food for different age groups are derived from the Chinese Dietary Guidelines (2022) [44], and the caloric coefficient of nine types of food is derived from the research results of Li et al. [45]. Protein, fat, and carbohydrate energy ratio data are from the Chinese food composition table (standard edition) released by the Chinese Nutrition Society [46]. The conversion coefficient used to calculate the actual demand and actual production of different types of food is based on the research results of Qian et al. [47] and Zhu et al. [48].

#### 2.2.2. Population Data

The demographic data for 2000, 2010, and 2020 are derived from the China Census Yearbook [49,50,51]. The demographic data of age, sex, and urban and rural populations for predictions of food demand are derived mainly from datasets published by Chen et al. [52] and Wang et al. [53] Since the historical demographic data and population structure under different scenarios (2011–2060) in the study area are highly correlated with the population structure under different scenarios (2011–2060) in Xinjiang (Table 1), based on the correlation coefficients presented in Table 1 and the predicted raster data of Xinjiang’s population by age and gender, RStudio software was utilized to calculate the population distribution by age and gender for both the baseline year and the most recent year.

### 2.3. Methods

#### 2.3.1. Food Production and Demand Calculation

Due to the shortage of land resources and water resources in oasis cities [54], the food production capacity in urban areas is relatively limited, and the grain produced in urban areas is used for seeds, feed, industrial processing, and reserve grain, which may cause food supply shortage and resource waste in urban areas. Moreover, China encourages the main grain-producing areas and main grain-selling areas to establish stable production-marketing relationships in various forms. Enterprises in the main grain-selling areas are encouraged to establish grain raw bases, processing bases and storage and logistics bases in the main production areas [55]. Therefore, for the UMA, this study does not consider regional grain reserves, seed grains, feed grains, or industrial grains, and assumes that the grains in the UMA are all used for regional consumption, so as to indicate the maximum food security capacity of the region. To explore the ability of regional consumption to the maximum extent by relying on its own production. The food production capacity is calculated using Formulas (1) to (4), where Formulas (1) and (2) calculate the energy production of each food and the total energy production of nine foods, respectively. The production of macronutrients (carbohydrates, protein, and fat) for each food and the total production of each macronutrient for the nine foods were calculated according to Formulas (3) and (4), respectively.

(1) $$TCP_{j}=FP_{j}\times CF_{j}\times CalF_{j\;}$$

(2) $$TCP=\overset{9}{\underset{j=1}{\sum }}TCP_{j}$$

(3) $$MNP_{j,q}=TCP\times EP_{j,\;\;q}$$

(4) $$MNP=\overset{9}{\underset{j=1}{\sum }}\overset{3}{\underset{q=1}{\sum }}MNP_{j,q}$$

In Formulas (1) to (4), *j* represents food groups, including grains, tubers, legumes, vegetables, meat, aquatic products, eggs, dairy products, and fruits. For convenience of later calculation, *j* is represented by numbers in this paper and the specific food groups represented by numbers are shown in Table A1 in Appendix A. *TCP_j_* is the total caloric production of food *j*, expressed in kcal. *FP_j_* is the production of food *j* in grams (g). *CF_j_* represents the conversion factor of food *j*, and *CalF_j_* is the caloric factor of food *j* (Table A1), which represents the number of calories in food *j*. *TCP* is the total caloric production of the nine foods. *EP_j,q_* represents the proportion of macronutrient *q* in food *j* (Table A2). *MNP_j,q_* is the yield of macronutrient *q* of food *j*, because macronutrients include carbohydrates, protein, and fat, three essential nutrients for human health, also known as productivity nutrients. Additionally, the macronutrients are numbered in this paper, where *q* represents the macronutrient number, and the specific meaning of the number is shown in Table A3 in Appendix A. *MNP* is macronutrient production, and it refers to nutrient production, which refers to the total carbohydrate production, total protein production, or total fat production of the nine foods in kilotons (kt).

For the calculation of food demand, UMA is located in a small ethnically populated area, and local residents mainly consume cereals, beef, and mutton, and they tend to consume high-calorie and high-fat foods [56]. Furthermore, with recent improvements in material living standards and health awareness, food consumption in the UMA has become increasingly important, and the dietary structure of residents is gradually diversifying [19]. Thus, this study divides urban food consumption patterns into actual consumption and dietary guideline-recommended structures when calculating food demand. The food demand is calculated using Formulas (5) to (8), wherein Formulas (5) and (6) calculate the total caloric demand of the nine foods and the caloric demand of the *j* food, respectively, while the macronutrient demand of the nine foods and the *q* macronutrient demand of the *j* food are calculated using Formulas (7) and (8), respectively.

(5) $$TCD_{j}=\overset{9}{\underset{a=1}{\sum }}Pop_{a}\times FI_{a,j}\times CalF_{j}$$

(6) $$TCD=\overset{9}{\underset{j=1}{\sum }}TCD_{j}$$

(7) $$MND_{q,j}=\overset{3}{\underset{q=1}{\sum }}\overset{9}{\underset{j=1}{\sum }}TED_{j}\times NRGP_{q,j}\times ECF_{q}$$

(8) $$MND=\overset{3}{\underset{q=1}{\sum }}\overset{9}{\underset{j}{\sum }}MND_{q,j}$$

From Formulas (5) to (8), *TCD_j_* is the total caloric demand of food *j*, a represents nine different age stages, *j* represents nine food types, Pop represents the population, *FI_a,j_* represents the demand for food j at age a (Table A4 and Table A5), and *CalF_j_* represents the caloric coefficient of food *j*. *TCD* is the total caloric requirement of the nine foods, measured in kilocalories (kcal). *MND_q,j_* is the macronutrient *q* requirement for food *j*. *NRGP_q_* refers to the energy ratio of macronutrient *q*, and *ECF_q_* is the energy conversion coefficient of macronutrient *q* (Table A3). *MND* is the macronutrient requirement for the nine foods, comprising carbohydrate, protein, or fat requirements in kilotons (kt).

#### 2.3.2. Food Production and Demand Balance Calculation

Food self-sufficiency is an important indicator used to measure food security. The Food and Agriculture Organization of the United Nations (FAO) gives a broad definition of food self-sufficiency: “The concept of food self-sufficiency is generally regarded as the degree to which a country can meet its food needs from its domestic production” [57]. However, in areas with restricted agricultural capacity, food self-sufficiency is defined as the ability of cities or regions to obtain sufficient food within their boundaries [27]. We use the food self-sufficiency index to evaluate the food production–consumption gap by combining the calculation methods of food self-sufficiency in published literature [23,58]. Although the assumption in this study that all food produced within the study area is consumed locally, thereby achieving food self-sufficiency within the region, has certain limitations, it holds practical significance for oasis metropolitan areas. This is because food production in oasis cities is highly dependent on water resources, and the distances between oasis cities are relatively long. Relying on long-distance food trade to meet food consumption not only incurs high environmental costs but also leads to certain nutritional and economic losses [35,36,37,38], negatively impacting the sustainable development of food systems in oasis cities. However, when oasis cities utilize limited resources to improve their own food production capacity and narrow the gap between food production and demand, their dependence on food trade for meeting food needs will decrease. In such a scenario, even if food trade is disrupted by unexpected risks, food supply will not collapse due to shortages. Furthermore, this carries significant practical implications for developing regional policies aimed at optimizing food production structures and advancing oasis urban agriculture while meeting food demand, which is consistent with the original purpose of our application of this indicator.

In this work, the food production-demand gap is measured mainly by the gap in total food calories, macronutrients and food diversity. The calculation formula is as follows:

(9) $$Gap=\frac{TCD-TCP}{TCP}\times 100\%$$

(10) $$\;\;\;\;\;\;\;\;\;\;\;=\frac{MND-MNP}{MNP}\times 100\%$$

(11) $$\;\;\;\;\;\;\;\;\;\;=\frac{TCD_{j}-TCP_{j}}{TCP_{j}}\times 100\%$$

In Formulas (9) to (11), *Gap* represents the food production-demand gap. In Formula 9, food caloric production and demand are obtained according to Formulas (2) and (6), respectively. In Formula (10), food nutrient production and demand are obtained from Formulas (4) and (8), respectively. In Formula (11), the demand for and production of food *j* are obtained via Formulas (1) and (5), respectively. If the gap is less than or equal to 0, local food production can meet the demand; however, if the gap is greater than 0, local food production cannot meet the demand, and it must be supplied by the surrounding region or long-distance transportation across the region; this also means that when the food supply chain is damaged or the food trade is blocked due to sudden emergencies, there is greater potential risk to regional food systems.

#### 2.3.3. Food Demand Forecast for the Future

In this study, the prediction of the future food demand of agricultural products is mainly based on the calculation of the total population in the shared socioeconomic path–typical concentration path (SSP–RCP) and the residents’ consumption structure (including historical consumption structure and the Chinese dietary guidelines consumption structure). The SSP–RCP is a new projection scenario proposed by the Sixth International Coupled Model Comparison Programme (CMIP6) of the World Climate Research Programme (WCRP), also known as ScenarioMIP. It was used in the Sixth Assessment Report of the IPCC 2022 (AR6) [59]. The SSP–RCP framework strengthens the consistency between future radiative forcing scenarios and shared socioeconomic scenarios [60,61] and has been widely applied to future land use change [62], future population prediction53, future ecosystem service value assessment [63,64], the prediction of future regional climate change [61,65], and other research fields (Table 2).

**Figure A1:**
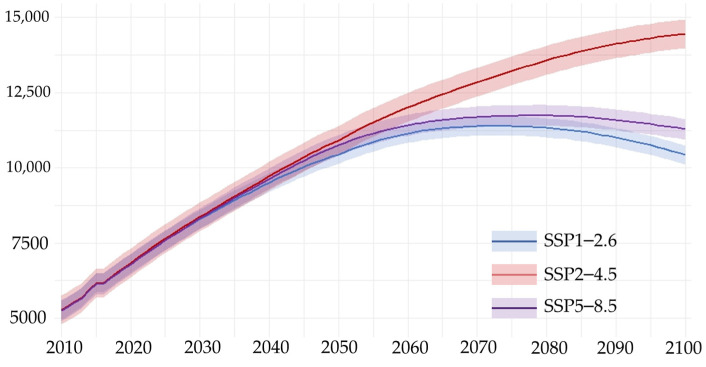
Population evolution of UMA in SSPs.

In this work, three coupled scenarios of shared socioeconomic paths and typical concentration paths (SSP–RCPs) were selected to predict the future change trend of the total population and the size of total population in different age groups of the Urumqi metropolitan area (Figure A1). The average population forecast data for 2011–2030, 2021–2040, and 2041–2060 are used as the basic-, near-, and mid-term populations respectively [65]. Since the population statistics and prediction of this dataset are raster datasets, this study obtains raster data in the study area through raster data clipping. Then, according to the correlation analysis results in Table 1, the food demand (including total calorie, macronutrient and food diversity demand) of the population in each age group in the basic-, near-, and mid-terms was calculated using Formulas (5)–(8). Food demand in the near- and mid-terms is compared with that in the base period to analyze the changes in food demand. Finally, the future food production-demand gap is calculated to clarify the pressure of future food demand on current food production capacity.

## 3. Results

### 3.1. Analysis of Food Production Capacity

#### 3.1.1. Total Caloric and Macronutrient Production

Overreliance on long-distance transportation and trade will correspondingly increase the negative effects of agroecological environments, such as carbon emissions. Thus, shortening the food supply chain can reduce food miles and effectively cope with short-term shocks by increasing the local food supply capacity, strengthening the resilience of the food system. In this study, the food production capacity of the UMA was measured by Formulas (1) to (4) in Section 2.3.1. The results (Table 3) revealed that total food caloric production increased significantly from 2000 to 2010 and showed a downward trend from 2010 to 2020; however, it was still higher than in 2000, and total food caloric production showed a fluctuating growth trend from 2000 to 2020. Plant source food energy production accounts for a relatively high proportion of total energy production; although animal source food caloric production is constantly increasing, the proportion is low. From 2000 to 2020, the highest proportion of total energy production from animal source food was only 14%. For macronutrients, the production of carbohydrates, proteins, and fats fluctuated and reached its highest level in 2010. From the protein point of view, high-quality protein can provide the human body with a complete proportion of essential amino acids and has a high digestible absorption rate of protein. The human body needs 20 amino acids to synthesize protein, of which 9 are essential amino acids (such as leucine, lysine, tryptophan, etc.), which must be ingested through the diet, so it is more important for human nutrition. According to the data, although the protein production in 2020 has declined compared with 2010, the proportion of high-quality protein production has reached 41%, an increase of about 4% compared with 2010, so the production level of protein nutrition is constantly improving. Overall, with urban development and the continuous adjustment of the agricultural production structure in the UMA from 2000 to 2010, the food production capacity of the UMA declined, but the food production structure was gradually optimized, and the nutrient supply capacity gradually improved.

#### 3.1.2. Food Diversity

The food production diversity in the food system within the Urumqi metropolitan area, including the production of cereals, potatoes, beans, vegetables, fruits, meat, eggs, milk and aquatic products has been constantly changing from 2000 to 2020, and the local production of cereals, vegetables, meat, and milk has fluctuated (Table 4). The production of fruits and eggs has steadily increased, and the fruit output in 2020 was 29 times that in 2000, with a relatively high growth rate. Mainly due to the improvement of agricultural technology and subsidies of agricultural economic policies, the fruit yield per unit area in the study area has significantly increased, and the planting area has significantly increased, resulting in a substantial increase in fruit yield. The changes in aquatic products first increased but then decreased, although overall they show an increasing trend. The production of tubers and legumes in 2020 was only 14.2% and 1.9% of the total production in 2000, respectively, mainly due to the accelerated urbanization of the Urumqi metropolitan area and the gradual adjustment of the agricultural planting structure and to ensure grain security, the planting area of tubers and legumes has been steadily declining.

### 3.2. Changing Characteristics of Food Demand

**Figure 2:**
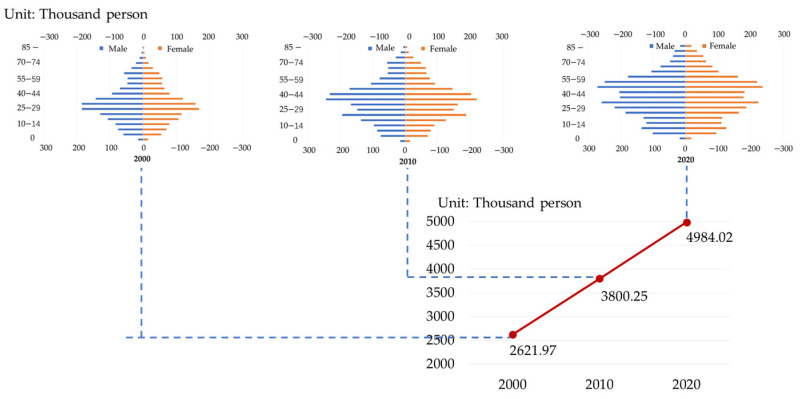
Total population and population pyramid of the UMA in 2000, 2010, 2020.

The UMA was in a stage of rapid urban development from 2000 to 2020. As shown in Figure 2, the population of the Urumqi metropolitan area continues to increase and presents a linear growth trend, with the total population increasing from 2,621.97 thousand to 4984.02 thousand. The annual growth rate is more than 110,000 (more than 100 thousand people), the newborn growth rate in the region is approximately 19%, and the growth rate of the female population aged 20–39 years is as high as 35%, far higher than the regional average level of Xinjiang (12%). In 2020, the total population of the Urumqi metropolitan area accounted for 41.33% of the total population of Xinjiang. Overall, the population in the core area of the Urumqi metropolitan area is growing rapidly, and the population is young, with a large population growth potential that will continue to increase in the future.

Based on the population changes, the food demand of the UMA is calculated using Formulas (5) to (8) in Section 2.3.1 (Table 5). From the perspective of total calorie consumption, with the continuous increase in population, residents’ food consumption in the UMA also continues to rise, and the total calorie demand of food consumption in 2020 was twice that in 2000. Residents’ carbohydrate consumption is within the range of dietary guidelines, but the consumption of protein and fat is greater than the guidelines’ recommendation (upper limit). This indicates that there is a nutritional imbalance in the food consumption structure of residents in the UMA, manifested mainly as high fat intake, especially of protein and fat of 122.9 kt and 126.4 kt, respectively, in 2020. This value was much higher than the dietary guidelines for protein (92.1 kt–109.2 kt) and fat (65.5 kt–97.1 kt).

To further clarify the food sources of the carbohydrate, protein, and fat intake of residents in the UMA, we illustrated the types of food consumed by the residents. The results (Table 6) revealed that the per capita consumption of the nine foods slightly changed from 2000 to 2010, while the consumption of all foods showed an increasing trend in 2020. For example, in 2020, the per capita consumption of fruit, eggs, and milk gradually increased, but it was still below the recommended dietary guidelines. The consumption of pulses was 16.8% below the lower limit of the dietary guidelines (90.8% in 2000) and of aquatic products was 31.6% below the lower limit of the dietary guidelines (74.7% in 2000), while the consumption of vegetables was within the recommended guidelines. Cereal and meat consumption was 11.0% and 4.9% higher than the upper limits of the dietary guidelines, respectively. These findings indicate that although the food consumption of residents in UMA is gradually becoming diversified, there is still a gap with the Chinese dietary guidelines’ recommendation. As Xinjiang is an autonomous region of ethnic minorities in China and one of the five important pastoral areas in China, the food consumption of Xinjiang residents often shows the characteristics of high grain and high meat consumption [66]. However, with the passage of time, the living standards of residents continue to improve, and meat consumption tends to gradually increase. The unique high meat consumption structure in Xinjiang has resulted in a serious deviation between the regional food consumption structure and the dietary guidelines.

### 3.3. Analysis of the Characteristics of Food Production and Demand

#### 3.3.1. The Gap Between Food Production and Current Consumption

**Figure 3:**
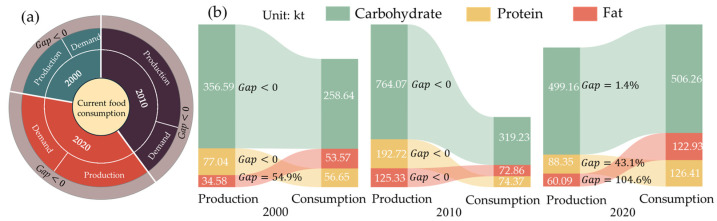
The food production-consumption gap of current dietary structure in the UMA in 2000, 2010 and 2020. ((**a**) is the total caloric food production-consumption gap. (**b**) is the macronutrient production-consumption gap).

Formulas (9) to (10) were used to analyze the pattern of food production and consumption of residents in the UMA. As shown in Figure 3a, there is no gap (Gap < 0) between total caloric production and consumption from 2000 to 2020, indicating that total caloric production is sufficient to meet demand. From the perspective of the gap between the production and consumption of macronutrients (Figure 3b), the production of carbohydrates, proteins, and fats in the food system in 2010 met the consumption needs of residents, whereas the gap between production and consumption of fats in 2000 was 54.9%. In 2020, the gaps for carbohydrates, proteins, and fats were 1.4%, 43.1%, and 104.6%, respectively. In addition, it also shows that, with the passing of time, the nutrient demand in the Urumqi metropolitan area is gradually higher than the nutrient supply capacity. Even in the case of sufficient total heat supply, residents in the UMA are still facing the risk of nutrient deficiency. This also shows that, due to the imbalance of food production, some food production is overproduced, some food production is too little, and then there is a total heat surplus and a lack of nutrition.

**Figure 4:**
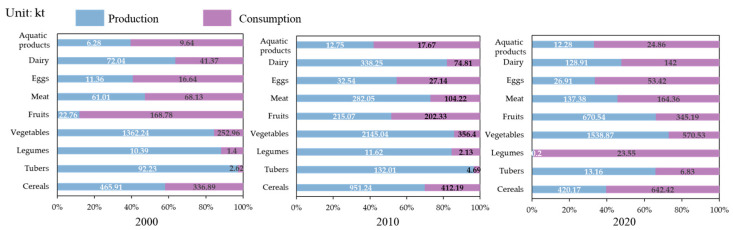
The food diversity gap for nine foods between the production and consumption of the current dietary structure in 2000, 2010, and 2020.

To further clarify the causes of nutritional imbalance in UMA, we calculated the production–consumption gaps of nine foods according to Formula (11) (Figure 4), which provided a scientific basis for optimizing the agricultural production structure and urban development planning in the UMA. In 2000 and 2010, the food production capacity was strong. Besides aquatic products, the production capacity of the nine foods can meet the consumption of residents, and the food supply capacity was relatively high. In 2020, the local production capacity of most food products appeared to be in a state of shortage; besides tubers, vegetables, and fruits, the local production capacity of other foods could not meet the consumption of residents, especially of legumes, which was extremely low. The results indicate that the risk of nutrient supply shortages among residents of the Urumqi metropolitan area in 2020 was primarily driven by a decline in the production of locally sourced animal-derived foods, such as meat, eggs, and milk, as well as plant-derived foods, including beans and grains. Since these food groups are major sources of protein, the production of protein-rich foods fell significantly short of meeting consumption demands. Consequently, the supply–demand gap for protein was substantially larger compared to that for carbohydrates and fats.

According to the results of Section 3.3.1 summary, from 2000 to 2020, the gap between local food production and the current consumption of residents in the Urumqi metropolitan area gradually increased, while the pressure on the urban food system increased, and self-sufficiency continued to decrease. In general, based on current consumption, the existing agricultural production structure layout of the Urumqi metropolitan area should be optimized to meet the current consumption demand of residents to the maximum extent, improving the food supply capacity of the Urumqi metropolitan area and the ability to cope with sudden risks to ensure the food safety of residents.

#### 3.3.2. The Gap Between Food Production and Dietary Guidelines for Food Consumption

**Figure 5:**
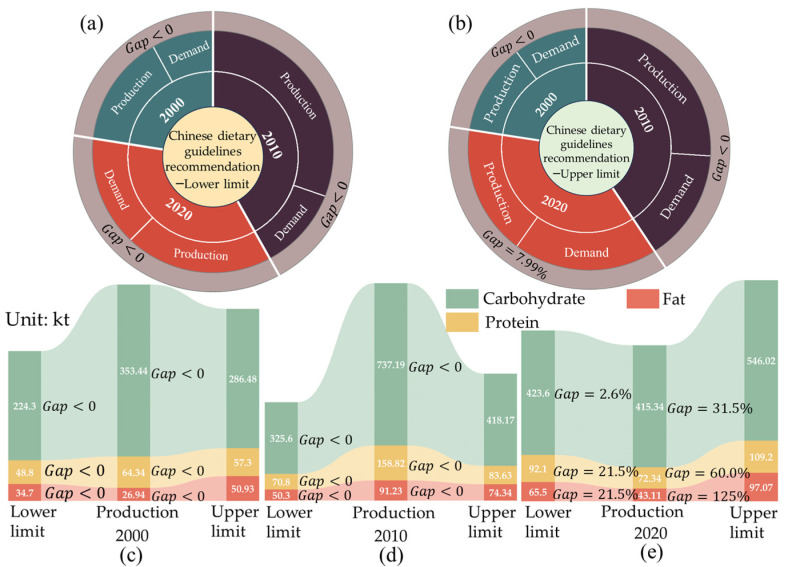
The gap between food production and food consumption of Chinese dietary guidelines in UMA in the 2000, 2010 and 2020 ((**a**) Total caloric gap between the production and lower limit of Chinese dietary guideline recommendation; (**b**) total caloric gap between the production and upper limit of the Chinese dietary guidelines’ recommendation; (**c**) the micronutrient gap between the production and lower and upper limits of the Chinese dietary guidelines’ recommendation in 2000; (**d**) the micronutrient gap between the production and lower and upper limits of the Chinese dietary guidelines’ recommendation in 2010; (**e**) the micronutrient gap between the production limit and lower and upper limits of the Chinese dietary guidelines’ recommendation in 2020.).

To further analyze the effect of food system localization production on urban food security, this paper analyzes the food system localization production–demand gap based on the residents’ dietary structure still following Chinese dietary guidelines in the future. Using Formulas (9) and (10), the characteristics of food production and demand under the food consumption model of food production and dietary guidelines were analyzed. The results show (Figure 5a,b) that, in terms of total calories, food production capacity can meet the recommended intake of Chinese dietary guidelines. However, over time, on the basis of the current food production capacity, if the local food production capacity of the UMA depends on the local food production capacity in 2020, it can meet only the lower limit of Chinese dietary guidelines recommendation, but there is a gap of approximately 7.99% in meeting the upper limit of the Chinese dietary guidelines’ recommendation for regional residents. Additionally, according to the results of the macronutrient production–demand gap assessment (Figure 5c,d), the gap of carbohydrates, protein and fat in 2000 was <0, indicating that carbohydrate, protein, and fat production in 2000 all met the nutrient requirements according to the dietary guidelines. The production of carbohydrates, proteins, and fats in 2020 was below the upper and lower limits of Chinese dietary guidelines recommendation. From the perspective of total caloric and macronutrient production and demand, when the residents of the UMA realized the Chinese dietary guideline consumption structure from 2000 to 2020, the ability of ensuring food security in the UMA gradually declined, and even if the total calorie intake of residents could be met, there would still be an imbalance in nutrient supply (Figure 5e). The total caloric production in 2020 met the lower limit of caloric intake recommended by the Chinese dietary guidelines, but there is a gap of 2.6%, 21.5%, and 21.5% between the production and the lower limit of the recommended intake of carbohydrates, protein, and fat, respectively, this represents that it must rely on external supply to achieve a balanced nutritional intake by residents in UMA.

**Figure 6:**
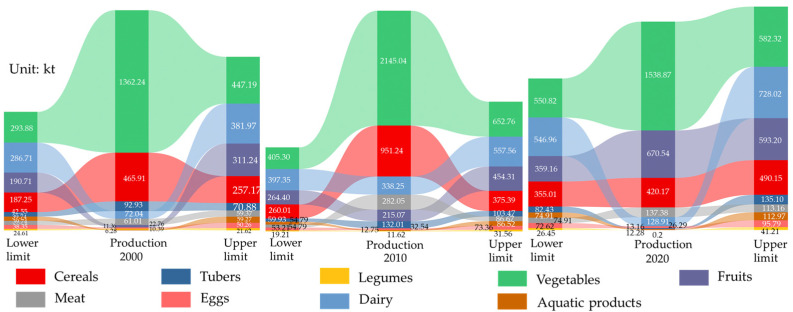
The food diversity gap for nine kinds of foods between the production and lower limit and upper limit of Chinese dietary guidelines consumption.

To further clarify the differences in the food nutrition sources of residents in the Urumqi metropolitan area, we calculated the differences in the production and demand of the nine food types using Formula (11). The results show (Figure 6) that the production of cereals, vegetables, meat, and fruits in 2020 met the upper limit of the recommended intake of the dietary guidelines; however, the production of potatoes, beans, eggs, milk, and aquatic products was far below the lower limit of the dietary guideline’s recommended intake, indicating the carbohydrate source is mainly cereals. Protein and fat mainly come from meat, plant protein, and egg; milk protein is relatively lacking, and the source of food is relatively simple.

According to the results of the Section 3.3.2 summary, the production of nine foods in the UMA cannot fully meet the intake recommended by Chinese dietary guidelines, thus, according to the current food production capacity of the UMA, the diversity of food sources and nutrient intake of residents cannot be fully met. Over time, the agricultural planting structure of UMA has been continuously adjusted, and its food production has been improved; however, the food production structure still cannot meet the food diversity demand under the dietary guidelines. The transformation and upgrading of residents’ dietary structures face problems such as insufficient food production and unbalanced food diversification. Affected by regional food production capacity, the transformation and upgrading of residents’ dietary structures face great challenges. Furthermore, based on a comparative analysis of the gap between food production, consumption, and the dietary guidelines (lower and upper limits), the gap between the actual consumption structure and production is small, indicating that the current food production capacity of the UMA can meet the demand of regional residents. This consumption structure of Chinese dietary guidelines should be the baseline for future food safety, and local food production capacity should be stabilized while the production of food diversity should be strengthened to meet the food needs of the population.

### 3.4. Prediction of Future Food System Stress

The future population changes in the UMA were clarified on the basis of the future population forecast dataset of Xinjiang under different SSPs (Figure A1). The average value of the historical consumption structure from 2019 to 2021 was recorded as the S0 dietary structure, the upper consumption limit of the dietary guidelines in 2022 was the S1 dietary structure, and the lower limit of residents’ dietary guideline consumption in 2022 was recorded as the S2 dietary structure. Future food consumption in the UMA under different dietary structures was calculated to indicate the total caloric and nutrient demand under different SSPs.

**Figure 7:**
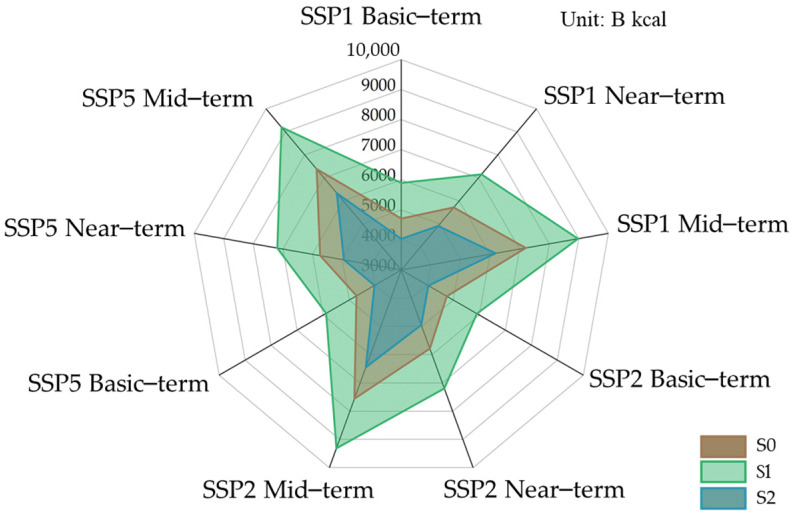
Total caloric consumption of three food consumption structures in basic-term, near-term and mid-term.

The results (Figure 7) reveal that in SSP1, SSP2, and SSP5, the total caloric consumption of the three consumption structures changed slightly during the basic-, near-, and mid-term, and the fluctuation reached 4.9%. Because the difference in population size is reflected mainly in different socioeconomic paths, this indicates that population change has little effect on total food consumption. From the perspective of food consumption structure, the total calorie consumption of residents in the UMA under the S1 consumption structure is the highest, and the total calorie consumption of S2 is the lowest, at 4035.43 B kcal in the basic-term, indicating that, in 2030, with the food production capacity unchanged (4044.8 B kcal in 2020), and residents follow the S2 consumption structure, it can rely on its own production capacity to achieve food security. In S0, total caloric consumption significantly increased in the medium term compared with the basic-term and mid-term, and the total caloric consumption of food will continue to increase as the population grows from 2020 to 2060. The growth rate of total caloric consumption of residents in the UMA shows similarities under different consumption structures, indicating that under the three consumption structures, the recent growth rate of total caloric consumption was between 21% and 22%, and the growth rate of total caloric consumption in the medium term was between 52% and 59% compared with the base period; this indicates that the residents’ dietary structure has a greater impact on food consumption. In the future, sustainable development should be the goal, with the lower limit of Chinese dietary consumption structure the “food security line”. The transformation and upgrading of residents’ dietary structures should be accelerated, which will improve the ability of the food system to cope with risks in the Urumqi metropolitan area.

**Figure 8:**
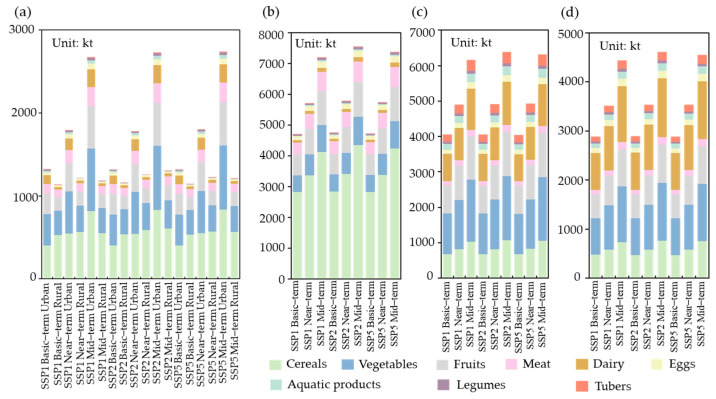
Nine kinds of food consumption in the SSPs ((**a**) Urban and rural food consumption in the historical consumption structure; (**b**) total food consumption in the historical consumption structure (S0); (**c**) lower limit of the Chinese guideline consumption structure (S1); (**d**) upper limit of the Chinese guideline consumption structure (S2)).

To further clarify the sources of dietary nutrition for future residents, we visualize the types of food consumed by residents in UMA. The results show (Figure 8) that, in the S0 consumption structure, residents’ dietary nutrition mainly comes from grains, meat and fruits, because the residents’ historical consumption structure was dominated by grain and meat consumption, which is much greater than that of grain and meat consumption under S1 and S2. Additionally, under the S0 consumption structure, there is a significant difference in food consumption between urban and rural residents. There is a significant increase in the consumption of the nine foods by urban residents, while the change in the consumption of the nine foods by rural residents is relatively stable. This indicates that the urbanization rate has a great impact on residents’ dietary structure and that accelerating the urbanization process may lead to a rapid increase in residents’ dietary consumption. In the future, residents’ understanding of dietary guidelines should be improved, and their awareness of dietary structure transformation should be strengthened, to ease the pressure on food demand caused by rapid regional urbanization.

We compare the future total food calorie demand under the three consumption structure models in Figure 7 with that in Table 3. A comparison of total food energy production in 2020 revealed that the food production and demand gap was 16.4–82.5% under the S0 consumption structure, 0–56.7% under the S1 consumption structure, and 45.8–127.4% under the S2 consumption structure. Moreover, we compared the future consumption of each food (Figure 8) with the production (Table 4), revealing that, under the three consumption structure patterns (S0, S1, and S2), except vegetables, the production capacity of the other eight foods is far from meeting future consumption requirements.

According to the above research results, in the future, with the continuous accumulation of the population in the metropolitan area, greater pressure will be exerted on the food production capacity in the UMA. Additionally, with the transformation of residents’ dietary structures, the diverse food demands of residents pose greater challenges to food production capacity. In the future, agriculture in the UMA needs to strengthen its self-production capacity and optimize the agricultural production structure. The use of local resources to build resilient food systems and enhance local food production and supply capacity should also strengthen the awareness of residents’ dietary structures and reduce residents’ deviation from the food consumption and dietary guidelines.

## 4. Discussion

From the perspective of total calories, macronutrients, and food diversity, this paper evaluated the gap between food production and demand in the UMA and reported that, under the actual consumption structure and the lower limit of the dietary guidelines, the annual performance from 2000 to 2020 was “total supply and demand balance, structural supply and demand imbalance”. In contrast, under the upper limit of Chinese dietary guideline consumption, from 2000 to 2020, performance gradually changed to “total supply and demand imbalance of structure”. Additionally, with the future growth of the urban population, the total demand for food will increase significantly, and the transformation of residents’ dietary structures will lead to higher requirements for food production capacity. Due to the restriction of water resources in the UMA [67], the food and other commodities production capacity is limited [68]; in order to address the “defining challenge of the 21st century” to feed the city sustainably, there is an urgent need to develop city–regional agriculture when local food production is insufficient to meet demand. So, it is important to accelerate the establishment of 1 and 1.5 h transportation networks in the UMA, strengthen the connection between UMA and Jimsar County, Qitai County and other food-producing counties, and build a diversified food supply system [21]. By these measures, the coupling distance of the urban food system should be appropriately reduced to improve the local food supply capacity [34], and local agricultural resources and the external food supply should be utilized to build a resilient oasis urban food system [45], improving regional food security and achieving sustainable development [69].

Additionally, our research results show that the pressure of changes in diet structure on local food production is much greater than that of population size, and residents’ dietary preferences are a more important factor affecting food safety. Meanwhile, previous research results also clearly indicate that the consumption of grain and meat in Xinjiang exceeds the upper limit recommended by China’s dietary guidelines [56]. There is some irrationality in Xinjiang residents’ food consumption structure, so it is the top priority to promote the transformation and upgrading of residents’ consumption structure. However, since the dietary structure is greatly affected by regional spatial differences, it is necessary to understand the residents’ dietary preferences and respect the residents’ dietary culture when conducting food selection education [56]. Therefore, we propose the formulation of regional dietary guidelines [45] based on the local dietary culture and agricultural production in Xinjiang. By optimizing the food production structure to promote the transformation of the consumption structure (“production to promote consumption”), it is also possible to promote the transformation of residents’ dietary structure to a more reasonable direction. Nevertheless, this study did not conduct an in-depth analysis of the intricate relationship between residents’ dietary preferences, regional dietary culture, agricultural production, and food safety, nor the influence of regional dietary culture on this dynamic interaction. Future research could further examine these complexities, exploring how food culture shapes dietary preferences, agricultural practices, and food security. Such investigations could provide valuable insights for the development of safe, healthy, and sustainable urban agriculture, as well as contribute to critical practices in sustainable agriculture and urban planning.

The findings of this study align with previous research, emphasizing the vital role of self-sufficient regions in enhancing regional environmental sustainability and minimizing transportation costs, particularly in areas with limited agricultural capacity, such as urban or metropolitan regions [11,35,36,70]. During short-term and sudden events, such as pandemics or trade disruptions, improvements in food self-sufficiency reflect the enhanced utilization of localized agricultural resources [71]. This, in turn, can mitigate the negative externalities associated with the production and transportation processes arising from the reliance of cities or metropolitan areas on external agricultural products [72]. This research underscores the importance of connecting cities to their surrounding areas through assessments of food self-sufficiency and specifically addresses food planning as an essential component of achieving sustainability in oasis metropolitan areas, consistent with prior studies [73]. Methodologically, this study shares similarities with previous research by employing a consumption- or demand-based model, which estimates the theoretical supply according to food consumption and dietary patterns, thereby analyzing food supply and demand within the region through the lens of self-sufficiency [17,74]. Importantly, this study introduces a novel approach by categorizing food production into caloric and nutritional production based on the caloric coefficient of different foods and different diet patterns. This approach enables the evaluation of food calorie and nutrient self-sufficiency levels, providing insight into the region’s food and nutritional security. Given the wide variety of nutrients produced in food systems, this study focuses solely on the production and consumption of macronutrients. However, for human health, the intake of micronutrients is equally important. In the future, the production and consumption of micronutrients could be incorporated into the evaluation framework for food self-sufficiency to comprehensively assess the capacity for regional nutritional self-sufficiency and to better clarify the level of regional food security. Based on the future population development of the region, this study evaluates the food demand in the near- and mid-term but lacks an assessment of food production capacity. Future studies should combine the constraints of food production (such as irrigation water and agricultural fertilizer inputs) and natural factors, such as disasters and climate change [75]. Future food production capacity should be further assessed [76], food demand should be comprehensively forecasted, and the gap between food production capacity and demand should be assessed to provide a more valuable reference for promoting the healthy and sustainable development of cities, optimizing food production layouts and coping with sudden risks [7].

The results of this study show that the adjustment of residents’ dietary structures will affect the food production situation, which is similar to the results of previous studies [76], but this study only emphasizes the importance of the change in regional residents’ consumption structures on the pressure of food production [77,78] and the development of food self-sufficiency in metropolitan areas and lacks empirical research on the impact of different consumption structures on the environment. Agriculture is an important source of greenhouse gas emissions [79], and the change in consumption structure plays an important role in reducing greenhouse gas emissions [80]. Follow-up studies should consider the impact of consumption structure on the environment, emphasize the importance of transformation and upgrade of residents’ diet structure for regional sustainable development from the perspective of environmental friendliness, and confirm the significance of food self-sufficiency for urban sustainable development from the empirical aspect.

Finally, although employing the food self-sufficiency indicator to assess the region’s food security in this study is feasible to a certain extent, certain limitations remain. This study merely highlights the environmental and economic impacts associated with urban food transportation and underscores the importance of investigating urban food self-sufficiency; however, it does not incorporate food transportation into the evaluation of food security. Future research should comprehensively assess both urban food self-sufficiency and food transportation. Subsequently, a holistic analysis encompassing regional food production, distribution, and consumption should be conducted to provide more comprehensive and targeted recommendations.

## 5. Conclusions

Based on empirical research, this paper identifies the degree to which food safety can be guaranteed by assessing the total calories and nutrients produced in the UMA and the food demands of its growing population. First, under the three current consumption structures, the dietary guideline’s upper and lower limits of consumption, current residents’ dietary consumption is characterized mainly by high caloric and high fat consumption. There is a significant gap between the consumption structure and that recommended by the dietary guidelines. If the residents’ consumption follows the S0 consumption structure, the pressure of food production in the UMA will increase significantly. Second, the gap between regional food production capacity and consumption has become increasingly significant, and there is a lack of diversity in the food supply and food nutrition sources. Finally, due to the influence of urban population growth and residents’ food consumption structure, the ability to guarantee future food security needs to be improved. Moreover, compared with population growth, food consumption structure exerts greater pressure on food production.

Therefore, it is necessary to expand the regional scope of the UMA to include major food-producing counties such as Qitai County and Jimsar County, which are close to the UMA, into the construction scope of the UMA, and improve the food supply of the surrounding areas to the urban center through the establishment of city-regional food circulation network. Furthermore, according to the sustainable use and effective planning of land, water, and other natural resources in the urban center and urban hinterland, improving the efficiency of resource production can maintain the food production capacity to a certain extent, and fundamentally narrow the gap between food production and consumption. Also, there is an urgent need to increase awareness of the transformation of residents’ dietary structures through food education, nutrition science popularization, and diet publicity, and, according to the regional dietary culture and agricultural production conditions and given the goal of meeting the nutritional and health needs of residents, the dietary structure of residents should be reasonably adjusted, and regional dietary guidelines should be formulated to increase public acceptance of dietary changes. and reduce the pressure on the regional food supply.

## Figures and Tables

**Table 1:** Population structure correlation between UMA and Xinjiang.

Date	Female (*p* Value)	Male (*p* Value)	Total Population (*p* Value)
2000–2020	0.904 ***	0.910 ***	0.907 ***
2011–2060 of SSP1	——	——	0.889 ***
2011–2060 of SSP2	——	——	0.999 ***
2011–2060 of SSP5	——	——	0.937 ***

*** indicates 0.01 significant level. *p* value is Pearson correlation coefficient.

**Table 2:** SSP-RCP scenarios.

Scenario	Category	Feature
SSP1-2.6	Low compulsion scenario	Sustainable development path (Low challenge)
SSP2-4.5	Moderate radiative forcing scenario	Middle path (Medium challenge)
SSP5-8.5	High compulsion scenario	Fossil energy development path (Mitigation challenges)

**Table 3:** Food production of UMA in 2000, 2010 and 2020.

Year	Total Caloric Production (TCP) Unit: B kcal			Macronutrient Production (MNP) Unit: kt		
	Plant Food	Animal Food	Total	Carbohydrate	Protein	Fat
2000	2677.10	189.14	2866.94	356.59	77.04 (22%)	34.58
2010	5142.27	816.00	5958.27	764.07	192.72 (37%)	125.33
2020	3634.46	410.34	4044.80	499.16	88.35 (41%)	60.09

Note: The numbers in parentheses indicate the proportion of high-quality protein. High-quality protein mainly comes from eggs, dairy, aquatic products, and meat such as egg, milk, fish, beef, poultry, mutton, and pork, and a diet consisting of foods from these high-quality protein sources is called a high-quality protein diet [44]. B kcal means billion kcal. kt is per thousand tons. The following is the same.

**Table 4:** Diversity of the food production of nine foods (Unit: kt).

Year	Cereals	Tubers	Legumes	Vegetable	Fruits	Meat	Egg	Dairy	Aquatic Products
2000	465.91	92.23	10.39	1362.24	22.76	61.01	11.36	72.04	6.28
2010	951.24	132.01	11.62	2145.04	215.07	282.05	32.54	338.25	12.75
2020	420.17	13.16	0.2	1538.87	670.54	137.38	26.91	128.91	12.28

**Table 5:** Current and Chinese dietary guidelines structure of food consumption in UMA.

Dietary Structure	Year	Total Caloric	Carbohydrate	Protein	Fat
Current dietary structure	2000	1710.2 B kcal	258.6 kt	53.6 kt	56.7 kt
	2010	2201.9 B kcal	319.2 kt	72.9 kt	74.4 kt
	2020	3574.6 B kcal	506.3 kt	122.9 kt	126.4 kt
Chinese dietary guidelines structure (Lower limit)	2000	1560.5 B kcal	224.3 kt	48.8 kt	34.7 kt
	2010	2264.9 B kcal	325.6 kt	70.8 kt	50.3 kt
	2020	2946.7 B kcal	423.6 kt	92.1 kt	65.5 kt
Chinese dietary guidelines structure (Upper limit)	2000	2291.8 B kcal	286.5 kt	57.3 kt	50.9 kt
	2010	3345.4 B kcal	418.2 kt	83.6 kt	74.3 kt
	2020	4368.1 B kcal	546.0 kt	109.2 kt	97.1 kt

**Table 6:** Per capita food consumption of UMA residents from 20002020 and per capita recommended consumption by the Chinese dietary guidelines 2022.

Food Type	Food Consumption of Current Dietary Unit: kg/Person			Chinese Dietary Guidelines Recommendation Unit: kg/Person	
	2000	2010	2020	Lower Limit	Upper Limit
Cereals	128.5	108.5	128.9	73.2	109.8
Tubers	1.0	1.2	1.4	18.3	36.6
Legumes	0.5	0.6	4.7	5.49	9.15
Vegetables	96.5	93.8	114.5	109.8	183.0
Fruits	64.4	53.4	69.3	73.2	128.1
Meat	26.0	27.4	33.0	14.64	27. 5
Eggs	6.4	7.1	10.7	14.64	18.3
Dairy	15.8	19.7	28.5	109.8	109.8
Aquatic products	3.7	4.7	10.0	14.64	27.5

## Data Availability

The original contributions presented in the study are included in the article, further inquiries can be directed to the corresponding author.

## Appendix A

**Table A1:** Calorie factors for the nine foods.

*j*	Type of Food	Conversion Factors (CF)	Calorie Factors (CalF)
1	Cereals	——	3.03
	Wheat	0.92	11.62
	Maize	1	——
	Rice	1	——
2	Legumes	1	2.12
3	Tubers	1	0.97
4	Vegetables	1	0.82
5	Meat	0.97	1.93
6	Aquatic products	0.57	1.16
7	Eggs	0.87	2.28
8	Milk	1	0.54
9	Fruits	0.86	1.62

**Table A2:** Proportion of nine food macronutrients.

Type of Food	Protein		Fat		Carbohydrate	
	Min	Max	Min	Max	Min	Max
Cereals	8%	12%	2%	2%	70%	70%
Legumes	19%	22%	15%	15%	0	0
Tubers	2.1%	2.6%	0.2%	0.2%	25%	25%
Vegetables	0	0	0	0	0	0
Meat	10%	20%	8%	30%	0	0
Aquatic products	15%	22%	1%	10%	1.5%	1.5%
Eggs	11%	13%	10%	15%	1.5%	1.5%
Milk	3%	3%	3%	4%	0	0
Fruits	0	0	0	0	5%	30%

**Table A3:** Macronutrient energy proportion and conversion factors.

*q*	Macronutrient	Energy Proportion Range (NRGP)	Energy Conversion Factors (ECF)
1	Carbohydrate	50~65%	4 kcal/g
2	Protein	10~15%	4 kcal/g
3	Fat	20~30%	9 kcal/g

**Table A4:** The lower limit of Chinese guidelines’ recommendations for nine foods at different ages (unit: g/d).

Age	Cereals	Tubers	Legumes	Vegetable	Fruits	Meat	Egg	Dairy	Aquatic Products
1–4	75	0	100	100	75	50	50	350	50
5–9	150	25	300	150	150	40	25	300	40
10–14	225	25	400	200	225	50	40	300	50
15–19	250	50	450	300	250	50	50	300	50
20–49	200	50	300	200	200	40	40	300	40
50–64	200	50	300	200	200	40	40	300	40
65–79	200	50	300	200	200	40	40	300	40
80 or over 80	200	50	300	200	200	40	40	300	40

**Table A5:** The upper limit of Chinese guidelines’ recommendations for nine foods at different ages (unit: g/d).

Age	Cereals	Tubers	Legumes	Vegetable	Fruits	Meat	Egg	Dairy	Aquatic Products
1–4	125	0	250	225	125	75	50	500	75
5–9	200	50	300	200	200	40	40	300	40
10–14	250	50	450	300	250	50	50	300	50
15–19	300	100	500	350	300	75	50	300	75
20–49	300	100	500	350	300	75	50	300	75
50–64	300	100	500	350	300	75	50	300	75
65–79	250	75	450	300	250	50	50	400	50
80 or over 80	250	75	450	300	250	50	50	500	50

Under different SSPs scenarios (Figure A1), the population development trend and total amount in the core area of the Urumqi metropolitan area are significantly different. The results show that, under the combined scenario of SSP1-2.6 and SSP5-8.5, the population in the core area of the Urumqi metropolitan area will show a trend of first increase and then decrease, and the total population will reach its peak around 2070. Under the SSP2-4.5 combination scenario, the population of the UMA will continue to grow rapidly, but the population growth will slow down in 2070.
